# Acute-phase proteins: As diagnostic tool

**DOI:** 10.4103/0975-7406.76489

**Published:** 2011

**Authors:** Sachin Jain, Vidhi Gautam, Sania Naseem

**Affiliations:** Department of Veterinary Pharmacology and Toxicology, College of Veterinary Science and Animal Husbandry, Jabalpur (MP) - 482 001, India

**Keywords:** Acute-phase proteins, C-reactive protein, diagnosis, haptoglobin, serum amyloid A

## Abstract

The varied reactions of the host to infection, inflammation, or trauma are collectively known as the acute-phase response and encompass a wide range of pathophysiological responses such as pyrexia, leukocytosis, hormone alterations, and muscle protein depletion combining to minimize tissue damage while enhancing the repair process. The mechanism for stimulation of hepatic production of acute-phase proteins is by proinflammatory cytokines. The functions of positive acute-phase proteins (APP) are regarded as important in optimization and trapping of microorganism and their products, in activating the complement system, in binding cellular remnants like nuclear fractions, in neutralizing enzymes, scavenging free hemoglobin and radicals, and in modulating the host’s immune response. APP can be used as diagnostic tool in many diseases like bovine respiratory syncytial virus, prostate cancer, bronchopneumonia, multiple myeloma, mastitis, *Streptococcus suis* infection, starvation, or lymphatic neoplasia. Thus, acute-phase proteins may provide an alternative means of monitoring animal health.

Animals undergoing external or internal challenge to their state of health mount a vigorous response including activation of both the innate and acquired immune systems. The innate immune system which covers those aspects of the host defense mechanisms not dependent on specific response, such as production of antibody, not only stimulates leukocyte activity but also effects many aspects of the host’s metabolic processes. The varied reactions of the host to infection, inflammation, or trauma are collectively known as the acute-phase response (APR) and encompass a wide range of pathophysiological responses such as pyrexia, leukocytosis, hormone alterations, and muscle protein depletion combining to minimize tissue damage while enhancing the repair process.[1] Another of these systemic responses to disease is an increase in the production by the liver of a number of plasma proteins which are known collectively as the acute-phase proteins (APP).[2–4]

The APR is a very complex reaction, involving local and systemic effects. One of these effects corresponds to changes in the concentration of some plasma proteins, mainly synthesized in the liver, which are called APP. The APR is induced by protein hormones called cytokines acting as messengers between the local site of injury and the hepatocytes synthesizing the APPs. Most cytokines have multiple sources, multiple targets, and multiple functions,[5] and they have been found in a large number of animal species including mammals, birds, fish, reptiles, and starfish.[6–10]

The changes in the concentrations of APPs are largely due to changes in their production by hepatocytes. The magnitude of the increases varies from about 50% in the case of C-reactive protein (CRP) and serum amyloid A (SAA). Under the influence of interleukin (IL), i.e., IL-1, IL-2, and tumor necrosis factor – alpha (TNF-α), liver cells synthesize and secrete APPs.

The maximum serum concentration of APPs is typically reached within 24 to 48 h after the initiation. A decline coinciding with the recovery from the infection is seen,[11] and generally, feed-back regulations will limit the response leading to its resolution within 4–7 days after the initial stimulus if no further stimulus occurs. When the receptor triggering has repeated pulses, the APR can become chronic.

Chronic inflammation (e.g., arthritis) can be perceived as a consecutive series of separate inflammatory stimuli. In such conditions, increased serum concentrations of APPs are generally observed.[12] However, the increase is lower than during acute episodes of inflammation or infection. There are also indications that the response to chronic compared to acute inflammation varies from one protein to another.[13]

The three most important APPs are CRP, serum amyloid P (SAP), and SAA.[14] Many APPs, such as CRP and SAA bind to microbial cell walls and they may act as opsonins and fix complement, thus promoting the elimination of microbes.

## Mechanism of Synthesis of Acute Phase Proteins

**Figure 1:**
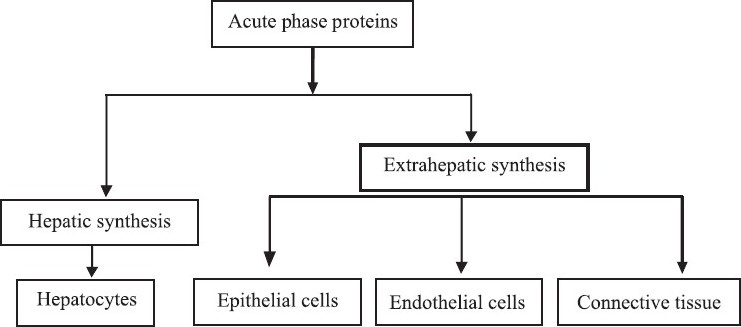
Synthesis of acute phase proteins

An APR is characterized, among other things, by fever and increases the numbers of peripherals leukocytes, in particulars, increasing the numbers of circulating neutrophils and their precursors. At the same time, cellular and biochemical alterations, in particulars the coordinated synthesis, of so-called APPs or APRs by hepatocytes take place in the liver [Figure 1].

## Regulation of Acute Phase Reactions and Synthesis of Acute Phase Proteins

According to Beutler and Cerami,[15] the APR is stimulated by the release of cytokines such as IL-1, IL-6, and TNF-α from macrophages and monocytes at the site of inflammatory lesions or infections.

Inflammatory cytokines such as IL-6, IL-1, TNF, and others such as transforming growth factor (TGF) and interferon (IFN) are produced by inflammatory cells. These proinflammatory cytokines induce local and systemic reactions.[16] These mediators are involved in cell activation of leucocytes, fibroblast, endothelial cells, and smooth muscle cells, result in a systemic release of cytokines, increase in the circulation of the cytokines, and then stimulate the hepatic APR.[17] Systemic reaction results in activation of the hypothalamus, reduction in growth hormone secretion, and a number of other physiological changes characterized by fever, anorexia, and catabolism of muscle cells.

TNF-α, IL-1β, and INF-γ are crucial for the expression of inflammatory mediators such as prostaglandins and leukotrienes and they induce the production of platelet-activating factor and IL-6. After stimulation by proinflammatory cytokines, Kuffer cells in the liver produce IL-6 and present it to the hepatocytes. Thus, IL-6 is the major mediator for the hepatocytic secretion of most of the APPs.

Activities are enhanced indirectly by activation of the pituitary/adrenal gland axis, which involves synthesis of adrenocorticotrophic hormone (ACTH) and subsequent production of cortisal. The increase in glucocorticoids during the APR is a result of cytokine stimulation of the pituitary–adrenal axis to produce adrenocorticotrophic hormone.[18] As a result, an increase in corticosterone, the main glucocorticoids, is observed later than the appearance of IL-6.[19] Cortisol can enhance expression of IL-6 receptors in liver cells and thus promotes IL-6-mediated synthesis of APPs.

Glucocorticoids are hormone that can be involved in APR as in mammals.[20] The role of glucocorticoids in birds seems to be both stimulatory and regulatory.[19] The administration of glucocorticoids to domestic fowl can also stimulate APP synthesis hormone,[18] which suggest that glucocorticoids may work independently of cytokines.

Negative regulatory loops can involve inhibition of synthesis of IL-6, IL-1, and TNF by cortisol and inhibition of the synthesis of IL-1 and TNF in monocytes by IL-6. Of all mediators participating in the induction and regulation of APP synthesis, IL-6 appears to induce the broadest spectrum of APPs whereas IL-1 and TNF only induce the synthesis of subsets of these proteins.

The mechanism for stimulation of hepatic production of APPs by proinflammatory cytokines has been extensively studied. Induction of the APPs by IL-1 following binding to the IL-6 receptor causes phosphorylation and degradation of inhibitor kappa B (IKB). The inhibitor of transcription factor nuclear factor kappa B (NF-kB) leads to the release of NF-kB and subsequent activation of acute-phase gene in nucleus.[21]

## Classification of Acute-phase Proteins

### On the basis of protein concentrations

#### Negative acute-phase proteins

The liver responds by producing a large number of APRs. At the same time, the production of a number of other proteins is reduced; these are therefore referred to as “negative” APPs. Negative APPs are albumin, transferring, transthyretin, transcortin, and retinol-binding protein.

#### Positive acute-phase proteins

Positive APPs are CRP, D-dimer protein, mannose-binding protein, alpha 1 antitrpysin, alpha 1 antichymotrypsin, alpha 2 macroglobulin, fibrinogen, prothrombin, factor VIII, von-Willebrand factor, plasminogen, complement factors, ferritin, SAP complement, SAA, ceruloplasmin (Cp), and haptoglobin (Hp).

Positive APPs serve different physiological functions for the immune system. Some act to destroy or inhibit growth of microbes, e.g., CAA and Hp. Others give negative feedback on the inflammatory response, e.g., serpins, alpha 2 macroglobulin and coagulation factors affect coagulation. Positive APPs are produced during the APR associated with anorexia and changed metabolism.

### On the basis of their mode of action

- APP classified as below: Protease inhibitors, e.g., alpha 1 antitrypsin, alpha 1 antichymotrypsin.
- Coagulation proteins, e.g., fibrinogen, prothrombin.
- Complement proteins, e.g., C2, C3, C4, C5, etc.
- Transport proteins, e.g., Hp, Cp, hemopexin.
- Other proteins, e.g., CRP, SAA, SAP, acid glycoprotein (AGP).

### Classification depending on the basis of their increased / decreased concentration in different species

The concentration of most of the APP increases, whereas other plasma proteins show decrease in their basal levels. Some APPs are present at very low concentration in normal state and may show increase up to 100 fold. This is the case of CRP or SAA in humans. Others increase between 2 to 10 times, whereas minor APPs are modified less than twofold. The APP pattern may vary from one species to another. As example, CRP that is a major APP in humans or dogs does not modify its concentration in cattle or cats.

In pig, a higher CRP serum concentration was observed in pigs with compared to without clinical signs of acute inflammation.[22] Other main APP in the pigs is CRP and Hp (increase of 8–10 and 2–10 times, respectively, in the turpentine model). SAA has also been described as a major APP and Cp is a minor APP in pigs. Beside albumin, fetuin and transferring are negative APPs (decreases of 20-40%).

In cattle, Hp and SAA are major APPs while fibrinogen, alpha-AGP, Cp, and alpha-antitrypsin are minor APPs in the cattle. SAA is the most studied APP in cattle. It can increase around 2–8 times during an APR and seems to react faster than Hp after the inflammatory stimuli.

In sheep, haptoglobin is a major APP in the sheep. Its concentration was raised up to 100 times after injection of yeast, whereas Cp and fibrinogen increased around four times, and albumin decreased. Increases of SAA of around 10 times normal values have been observed in ewes with mastitis induced experimentally. SAA also increased in milk.

In dogs, the behavior of CRP is similar as in humans. The concentration of CRP dramatically rises from undetectable levels to around 100 mg/mL in the first 24 h after surgery, declining after that. Hp, alpha-AGP, and ceruplasmin increased moderately (around twofold), and the concentration of alpha-antitrypsin was not modified.[23]

### General role of APPs in the body

The function of positive APPs is regarded as important in optimization and trapping of microorganism and their products, in activating the complement system, in binding cellular remnants like nuclear fractions, in neutralizing enzymes, scavenging free hemoglobin and radicals, and in modulating the host’s immune response. CRP is the first described APP in 1930. It binds directly to several microorganisms, and activates the complement system by the classical C1q pathway, and acts as opsonins. SAA was first described in 1994 and is an apolipoprotein of high-density lipoproteins (HDL).

An APP is thought to influence HDL–cholesterol transport. In tissues, it attracts inflammatory cells and inhibits the respiratory burst of leukocytes.[24] It is also described to bind lipopolysaccharide (LPS), comparable to LPS binding protein. Hp strongly binds to hemoglobin, has anti-inflammatory capabilities, and binds to integrins on leukocytes. Although representing a positive APP, its quantity may decrease on massive erythrolysis and when blood is hemolytic. Cp has histaminase, ferroxidase activity, and scavanges Fe^2+^ and free radicals, while α_2_ macroglobulin (α_2_MG) binds to proteolytic enzymes [Table 1].

**Table 1:** Biological activities of selected acute phase proteins

Acute phase protein	Biological activity
Haptoglobin	Binds with hemoglobin
	Bacteriostatic effect
	Stimulation of angiogenesis
	Role in lipid metabolism/development of fatty liver in cattle
	Immunomodulatory effect
	Inhibition of neutrophils respiratory burst activity
C-reactive protein	Complement activation and opsonization
	Modulation of monocytes and macrophages, cytokine production
	Binding of chromatin
	Prevention of tissue migration of neutrophils
Serum amyloid A	Transport of cholesterol from dying cells to hepatocytes
	Inhibitory effect on fever
	Inhibitory effect on the oxidative burst of neutrophilic granulocytes
	Inhibitory effect on *in vitro* immune response
	Chemotexic effect on monocytes, leukocytes, and T cells
	Induction of calcium mobilization by monocytes
	Inhibition of platelet activation

### Pattern recognition molecules, pentraxins, and C-reactive proteins

In the past, the innate immune system was considered to be a primitive static system; nowadays delve into its complexity. It is a system that is able to recognize and respond to danger signals represented by a limited number of highly conserved structures of microorganisms [pathogen-associated molecular patterns (PAMPs)] and several cell products associated with a breach in defenses. For this purpose, the innate immune system possesses a large number of soluble (e.g., pentaxins), membrane-bound [e.g., toll-like receptors (TLR)], and cytosolic (e.g., nod-like receptors) “receptors.”[25] They are known collectively as pathogen recognition receptors, or pattern recognition receptors, but a more accurate term is pattern recognition molecules (PRMs).

Pentraxins are superfamily of proteins, phylogenetically conserved from arachnids to mammals and characterized by the presence of their carboxyl and terminal of a 200 amino acid pentraxin domain. The pentraxin was first assigned to CRP for its ultrastuctural appearance of five subunits. These protein pentraxins have been around in the animal kingdom for some time, since a closely related homolog, limulin, is present in the hemolymph of the horseshoe crab, not exactly a close relative of *Homo sapiens*. Human CRP is composed of five identical polypeptide units noncovalently arranged as a cyclic pentamer around a Ca-binding cavity. Based on the primary structure of the subunits, the pentraxin are divided into short and long pentraxins. The short pentraxins reactive proteins and serum amyloid pentraxins component are produced by liver and represent the main APPs in human and mouse, respectively. The long pentraxins, i.e., PTX3, are produced by innate immunity cells [e.g., polymorphic mononuclear cells (PMN), macrophages, and dendritic cells], interact with several ligands, and play an essential role in innate immunity.[26] PTX3 provides a paradigm for mode of action of humoral innate immunity. Thus, pentraxins recognize a wide range of exogenous pathogenic substances and altered self-molecules and in species-specific manner behave as APPs.[27]

A major property of CRP is its ability to bind in a Ca-dependent fashion, as a pattern recognition molecule, to a number of microorganisms which contain phosphorylcholine in their membranes, the complex having the useful property of activating complement. This results in the deposition of C3b on the surface of the microbe which thus becomes opsonized (i.e., made ready for the table) for adherence to phagocytes.[28]

CRP was originally discovered by Tillett and Francis[29] in 1930 as a substance in the serum of patients with acute inflammation that reacted with the C-polysaccharide by the liver and by adipocytes.

#### Functions

CRP levels rise dramatically during inflammatory processes occurring in the body. CRP rises up to 50,000 fold in acute inflammation, such as infection. It rises above normal limits within 6 h, and peaks at 48 h. CRP binds to phosphorylcholine on microbes. It is thought to assist incomplete binding to foreign and damaged cells and a cell enhances phagocytosis by macrophages, which express a receptor for CRP. It is also believed to play an important role in innate immunity, as an early defense system against infections.

#### Diagnostic use

CRP is used mainly as a marker of inflammation and infection. Measuring and charting CRP values can prove useful in determining disease progress or the effectiveness or treatments. Viral infections tend to give a lower CRP level than bacterial infection.

#### Role in cardiovascular disease

Patients with elevated basal levels of CRP are at an increased risk of diabetes, hypertension, and cardiovascular disease. CRP can exacerbate ischemic necrosis in a complement-dependent fashion and that CRP inhibition can be a safe and effective therapy for myocardial and cerebral infarcts; this has only been demonstrated in animal models.

#### Diagnostic test

Various analytical methods are available for CRP determination, such as enzyme linked immunosorbent assay (ELISA), immunoturbidimetry, rapid immunodiffusion, and visual agglutination. To measure the CRP level, a “high-sensitivity” CRP or hs-CRP test needs to be performed and analyzed by a laboratory. This is an automated blood test designed for greater accuracy in measuring low levels of CRP, which allows the physician to assess cardiovascular risk. If a result in the low-risk range is found (<1 mg/L), it does not repeating. Higher levels need repeating, and clinical evaluation as necessary.

### Relevance of genetic vs environmental determinants of CRP

Elevated plasma levels of CRP are associated with increased risks of ischemic heart disease and ischemic cerebrovascular disease.[30–33] The random assortment of genes that occurs during gamete formation provides a relatively unbiased method of assessing whether risk factors that have a genetic component are in fact causally related to clinical outcomes. This phenomenon has sometimes been termed “mendelian randomization.” Thus, genetic variants that specifically increase plasma levels of CRP[34,35] provide an ideal system to assess the consequences of lifelong high CRP levels, independently of other risk factors.[36]

According to the study conducted by Zacho *et al*.,[37] on genetically elevated CRP and ischemic vascular disease showed that CRP genetic variation was associated with elevated CRP levels without predicting an increased risk of ischemic vascular disease. Genetic variants that are associated with lifelong increases in plasma CRP levels are not associated with an increased risk of ischemic heart disease or ischemic cerebrovascular disease. The increase in the risk of ischemic vascular disease associated with higher plasma CRP levels observed in epidemiological studies may not be causal, but rather that increased CRP levels are simply a marker for atherosclerosis and ischemic vascular disease.

### Serum amyloid A

Serum amyloid A (SAA) proteins are a family of apolipoproteins and produced by the liver. These proteins play a highly essential role in all animals. Acute phase SAA proteins (A-SAAs) are secreted during the acute phase of inflammation. These proteins have several roles, including the transport of cholesterol to the liver for secretion into the biles, the recruitment of immune cells to inflammatory sites, and the induction of enzymes that degrade, such as amyloideosis, atherosclerosis, and rheumatoid arthritis. Several isotypes of SAA are found; types 1 and 2 represent positive APPs. In the bovine, also a negative protein crossreacting with anti-SAA serum has been described.[38] The acute phase SAA isoforms have been reported in mice, called SAA1, SAA2, and SAA3.

Besides the acute phase SAAs, constitutive variants are described.[39] Human SAA4 is normally present in serum.[40,41] Rabbit SAA3[42] is formed by synoviocytes, fibroblasts, and macrophages, and is not a blood protein. The mammary gland is a well-known source of an SAA3 variant[43–45] occurring in colostrum and in mastitis milk that should have beneficial functions for the gut mucosa of the offspring.[46–48]

### Haptoglobin

Haptoglobin (Hp) is a protein in the blood plasma that binds free hemoglobin released from erythrocytes with affinity and thereby inhibits its oxidative activity. The haptoglobin–hemoglobin complex is used to screen for and monitor intravascular hemolytic anemia.

#### Clinical significance

Haptoglobin is produced mostly by hepatocytes but also by other tissues, e.g., skin, lung, and kidney. Reticuloendothelial system will remove the haptoglobin–hemoglobin complex from the body; haptoglobin levels will be decreased in hemolytic anemia. In the process of binding hemoglobin, haptoglobin sequesters the iron within hemoglobin, preventing iron-utilizing bacteria from benefiting from hemolysis. Haptoglobin is ordered whenever a patient exhibits symptoms of anemia, such as pallor, fatigue, shortness of breath, along with physical signs of hemolysis, such as jaundice or dark-colored urine.

Decreases in haptoglobin can support a diagnosis of hemolytic anemia, especially when correlated with a decreased RBC count, hemoglobin and hematocrit, and also an increased reticulocyte count. If the reticulocyte count is increased but the haptoglobin level is normal, this may indicate that the cellular destruction is occurring in the spleen and liver, which may indicate a drug-induced hemolysis or a red cell dysplasia.

The spleen and liver recognize an error in the red cell and destroy the cell. This type of destruction does not release hemoglobin into the peripheral blood, so the haptoglobin cannot bind to it. Thus, the haptoglobin will stay normal. If there are symptoms of anemia, it is most likely not due to hemolysis but instead some other error in cellular production, such as aplastic anemia. Haptoglobin levels which are decreased but do not accompany signs of anemia may indicate liver damage, as the liver is not producing enough to begin with [Table 2].

**Table 2:** Haptoglobin level in various species

Species	Normal range (Mg/dL)	Increase in APR (Mg/dL)
Bovines	0.0–0.5	1.0–3.0 and <
Canines	0.3–3.6–5	4.0–9.0
Porcine	0.0–2.2	3.0–8.0
Felines	0.7–2.0	3.0–10
Ovine	0.0–1.0	0.0–3.0
Humans	1.0–3.0	4.3–7.8

### Mannose-binding protein

The most important acute phase opsonin is the Ca-dependent mannose-binding protein (MBP), which can react not only with mannose but several other sugars, so enabling it to bind with an exceptionally wide variety of Gram-negative and -positive bacteria, yeasts, viruses, and parasites; its subsequent ability to trigger the classical C3 convertase through two novel associated serine proteases (MASP-1 and MASP-2) qualifies it as an opsonins. MBP is a multiple of trimeric complexes, each unit of which contains a collagen-like region joined to a globular lectin-binding domain. This structure places it in the family of collectins (collagen + lectin) which have the ability to recognize “foreign” carbohydrate patterns differing from “self” surface polysaccharides normally decorated by terminal galactose and sialic acid groups, while the collagen region can bind to and activate phagocytic cells through complementary receptors on their surface.[49] Collectins are a group of proteins containing C-type carbohydrate recognition domains (CRD) attached to collagen-like regions via a-helical coiled-coil regions.[50] The group includes mannan-binding lectin (MBL), surfactant proteins A and D (SP-A and SP-D), conglutinin, 43-kDa collectin (CL-43), and the recently identified CL-L1 and CL-P1.[51,52] Conglutinin and CL-43 have so far only been identified in the *Bovidae*. MBL, conglutinin, and CL-43 are plasma proteins synthesized in the liver. The collectins play an important role in the nonadaptive immune defense, as demonstrated by the finding that SP-A- or SP-D-deficient mice are susceptible to a variety of infections.[53,54]

The collectins, especially MBP and the alveolar surfactant molecules SP-A and SP-D, have many attributes that qualify them for a first-line role in innate immunity. These include the ability to differentiate self from nonself, to bind to a variety of microbes, to generate secondary effector mechanisms, and to be widely distributed throughout the body including mucosal secretions. MBL is the only collectin that activates the complement system. After binding to microorganisms, the MBL-associated serine proteases cleave and activate C4, C2, and C3.[55] This may lead directly to complement-mediated lysis of the microorganisms or may indirectly increase the opsonization mediated by deposition of C3.

MBP is an APR, and its deficiency is associated with the common opsonic defect and susceptibility to infections and atopic constitution. The high concentration of MBP in infants may best be explained by exposure to novel environmental antigens in early childhood, which suggests a protective role for MBP during the period of immaturity of the immunosystem. In older children, the high level of MBP can probably be explained by childhood infections and the ensuing need of MBP.[56]

### Role of phycolins and collectins as acute phase proteins

When infection exceeds the capacity of the local cells and mediators for containment and/or elimination of an organism in a tissue site, a systemic host response can ensue. This response involves release of numerous APPs from the liver in response to pathogen products (e.g., endotoxins) and cytokines (e.g., IL-1, TNF-α, and IL-6 generated locally and systemically). The liver produces complement, collectins, and pentraxins together with numerous other classes of molecules involved in host defense, inflammation, clotting, cardiovascular function, and so forth. Probably because of the presence of repeated and severe infections, chronic obstructive pulmonary disorder (COPD) is characterized by the elevations of APPs, including CRP.[57,58] Systemically, these molecules may contribute to disease, because they can have inflammatory actions caused by activation of leukocytes and activation of complement. Locally, however, the antimicrobial effects of opsonins are likely to be protective. There is a growing realization that local cells in the airways can produce collectins and APPs, including complement proteins and pentraxins.[59–63] Components of this local APR appear to be induced by cytokines and TLR ligands.[59] Pentraxins recognize a wide range of exogenous pathogenic substances and altered self-molecules and in species-specific manner behave as APPs.

A recent study showed that CRP is highly expressed by airway epithelium and that CRP in sputum and nasal lavage fluid is capable of killing bacteria.[63] Future studies are needed to determine the relative importance of local and systemic APRs in host defense in the airways. A newly recognized family of molecules, the intelectins, has been identified and may play a role similar to that of pentraxins and collectins in the airways.[64]

### Transferrins

Transferrin is a blood plasma protein for iron ion delivery. Transferring is a glycoprotein, which binds iron very tightly but reversibly.

Transferrin is also associated with the innate immune. Transferrin is found in the mucosa and binds iron, thus creating an environment low in free iron, where few bacteria are able to survive. The levels of transferrin decrease in inflammation, seeming contradictory to its function. A decrease in the amount of transferrin would result in hemosiderin in the liver. Transferring has a bactericidal effect on bacteria, in that it makes Fe^3+^ unavailable to the bacteria.

A transferrinemia is characterized by anemia and hemosiderosis in heart and liver. The iron damage to the heart can lead to heart failure. The anemia is typically microcytic and hypochromic (red blood cells are abnormally small and pale).

## APPs in Veterinary Diagnosis

### Bovine respiratory syncytial virus

The sperm concentrations reached for SAA and haptoglobin during the BRSV-induced APR were generally the same or higher than bacterial infections in calves. The magnitude and the duration of the haptoglobin response was found to correlate well with the severity of clinical signs (fever) and with the extent of lung consolidation while SAA responded most rapidly to infection.[65]

### Prostate cancer penitents with bone lesions

Prostate cancer has a propensity to metastasize to the bone. Correctly, there are no curative treatments for this stage of the disease. Sensitive biomarkers that can be monitored in the blood to indicate the presence or development of bone metastases and/or response to therapies are lacking. The cluster of unique proteins in the sera of patients with bone metastases was identified as isoforms of SAA.[65]

### Hematological and neoplastic diseases of the dog

Serum concentrations of APPs, Hp, Cp, SAA, and CRP were determined in healthy dog and dogs with different diseases, grouped as acute inflammation, hematological neoplasias [hemotologic tumor (HT)], including epithelial, mesenchymal, and mixed and autoimmune hemolytic anemia. Measurement of APPs may be helpful to assess clinical evolution and monitor treatment of these processes.[66]

### Growing calves suffering from bronchopneumonia under filed conditions

Blood samples were taken from calves with respiratory disease the first day of examination for determination of the serum concentration of haptoglobin, fibrinogen, α-2- and γ-globulins, and albumin. The two serum proteins haptoglobin and fibrinogen, and especially haptoglobin, were useful for the identification of calves requiring an anti-inflammatory treatment.[67]

### Multiple myeloma

Long-lasting APR occurs in patients with chronic inflammation and cancer. IL-6 was negatively correlated with five out of nine (C1-INH, C8, C9, AGP and haptoglobin) positive APPs, but positively correlated with CRP.[68]

### Endotoxin mastitis

A crossover study was conducted to investigate the effect of intramammarily infused lipopolysaccharide (LPS) on the APR in early (EL) and in late (LL) lactation. Nine cows received intramammary injections of 100 *µ*g of *Escherichia coli* LPS during EL and LL. The milk TNF-α is on average higher in EL. SAA concentration was not correlated being on average higher in EL. SAA concentration was not correlated with changes in milk appearance.[69]

### Mastitis

In a well-managed dairy herd, in addition to clinical mastitis, subclinical mastitis should be efficiently detected. The most promising parameters for monitoring subclinical mastitis are milk *N*-acetyl-D-glucosaminidase activity, lactose and electrical conductivity, along with some other indicators such as optical and milk flow measurements, preferably with an interquarter evaluation included in the test. APPs, Hp, and SAA are also potential candidates for mastitis monitoring.[70]

The concentration of Hp in serum has been shown to dramatically increase in cows with experimental and spontaneous coliforms mastitis. The first APPs measured from milk and used as indicators of inflammation are bovine serum albumin and α-1 trypsin inhibitor. Hp and SAA were measured from milk and serum, and compared as tests to detect intramammary infection (IMI). A significant correlation was found between the concentrations of Hp in the serum and milk, but the concentrations of SAA in the serum and milk were not related. No correlation was found between Hp and SAA levels in milk. SAA could distinguish between mild and moderate mastitis.

Using a threshold value of 0.02 mg/mL for milk Hp and 0.55g/mL for milk SAA, both tests has a high specificity (100%) with no false positive results, and a reasonable sensitivity[71,72] for the diagnosis of mastitis. Hp and SAA concentrations below the detection limit were considered as good indicators of healthy udder quarters. A substantial variation in Hp and SAA concentrations in milk was observed in udder quarters with chronic subclinical mastitis.[73] The CRP is not regarded as an APP in cattle, but has been tested as an indicator for mastitis. The concentration of CRP was shown to increase in bovine milk during mastitis. The capacity of milk CRP to distinguish between healthy and mastitic quarters was found to be poor, and the correlation between the concentration of the CRP in milk and somatic cell count (SCC) was low (*r*=0.32). It seems that the CRP does not have the best potential to be used in the detection of mastitis.[70]

SAA and Hp for the detection of bovine mastitis clinical and subclinical mastitis can be revealed by high serum concentrations of Hp and SAA. It is also of interest that the concentration of APPs in milk from infected quarters is higher than that in noninfected quarters. By testing milk, a large number of samples are easily obtained in a way that is less stressful than obtaining a blood sample. If APPs are produced locally in the udder as a response to mastitis, they might be more rapid and sensitive markers of acute inflammation than the somatic cell count. However, future studies on the applicability of APP in milk as markers of mastitis are needed.

### *Streptococcus suis* infection in the pig

In order to measure serum transthyretin (TTR) in the pig during an APR, an assay was developed using anti-human TTR antibodies which crossreacted with porcine TTR. Following *Streptococcus suis* type-2 infection TTR showed a negative APR with serum concentrations reaching a significantly lower level at 2 days following infection.[74]

## Starvation

Negative reacting proteins are normally present in healthy animals, but will decrease in concentration due to the APR. Albumin is generally accepted as negative APP present in most species. The negative reacting protein transferrin is possibly involved in the innate immunity, perhaps by sequestering ferric ions to prevent pathogens and parasites from using nutrients. Retinol-binding protein (RBP) is a small-molecular-weight protein which is the exclusive protein for the transport of vitamin A (retinol) in the body. The synthesis and secretion of RBP by parenchymal hepatocytes is mainly controlled by the combination with the larger, tetramer protein, transthretin. The complex formation appears to be necessary to prevent extensive loss of the low-molecular-weight RBP through glomerular filtration.[75]

During starvation, there is no full positive response, and a general depression of hepatic protein synthesis occurs. Malnutrition and the anorectic effects of pro-inflammatory cytokines in the brain result in a negatively changed hepatic synthesis. The major three of these cytokines (TNF-α, IL-1, and IL-6) have a profound behavioral, neuroendocrine, and metabolic effect.[76–79]

Moreover, there is evidence that cytokines and their cognate receptors are present in the neuroendocrine system and brain. In laboratory animal species, IL-1, IL-6, and TNF-α have been found to modulate intermediary metabolism of carbohydrate, fat, and protein substrates, regulate hypothalamic–pituitary outflow, and act in the brain to reduce food intake.[76,78]

On starvation and negative energy balance associated with most diseases, muscle proteins are catabolized for amino acid supply of the hepatic APP formation and as source of energy. Especially for those APPs which rapidly and quantitatively increase in blood, their formation may have amino acid impact. An increased hindquarter protein catabolism exceeding the hepatic protein synthesis, and efflux of glutamine and alanine from the hindquarter was measured during a porcine-induced endotoxemia study.[80] For growth during and after recovery from a disease, food requirements for amino acids thus may differ from the formula in ordinary food. Some pig studies indicate positive influences of additional dietary tryptophan[81] or L-arginine.[82]

## Lymphatic Neoplasia

Median CRP concentration was increased in all groups with neoplastic lymphatic disorders like lymphomas, malignant lymphoma, and multiple myeloma. Hp level was specially increased in dogs with acute lymphoblastic leukemia (ALL) and malignant lymphoma. The median values in the dogs with ALL were significantly higher than in dogs with other neoplastic lymphatic disorders.[83]

### APPs in dogs and cats: Current knowledge and future perspectives

The APR and clinical application of monitoring APPs in dogs and cats include proper and adequate clinical interpretation. In addition, the diagnostic use of APPs and their possible application in monitoring treatment can be considered as one of the most interesting and promising practical applications of these proteins. New and cheaper automated assays for determination of the main APPs in small animals will contribute to a wider use of these proteins as biomarkers of infection and inflammatory lesions.[80]

### Some application of acute phase proteins as diagnostic tool in animals

APP is applied as unspecific markers of clinical and subclinical infections, to discriminate between acute and chronic disease and for prognostic purposes, since the duration and magnitude of the response reflect the severity of the disease and the effect of treatment.

### Haptoglobin: A marker of herd health status in pigs

Canadian and American researchers showed that in immunologically naive boars moved to new facilities, an increase in Hp concentration was observed before the clinical signs of the disease were evident.[84]

The Hp concentration remained high, and the animals subsequently showed clinical signs of the disease (depression, respiratory distress, and cyanosis). Higher Hp serum concentration prior to the clinical signs could be due to early, subclinical pathological conditions. Also, lower gaining pigs were found to have higher Hp levels than gaining pigs. The serum Hp concentration increased significantly with age in conventional slaughter pigs without clinical signs but not in slaughter pigs from high health (SPF-X) herds, indicating that subclinical disease in conventional herds may be the cause of higher serum Hp concentration in older pigs. Therefore, Hp seems to be a promising marker of health status by reflecting a broad spectrum of ongoing clinical as well as subclinical diseases.

### Serum amyloid A as a prognostic marker in equine respiratory disease

SAA is useful in the management of bacterial and viral infections in horses by large-scale monitoring in stables and as the prognostic tool in relation to clinical severity and the recovery of individual horses. Serum concentrations of SAA have been found to increase in foals during infection with *Rhodocoocus equi*, equine influenza serotype A2 (H3N8), equine herpes virus serotype 1, and *Streptococcus equi*. A statistically significant association between SAA serum concentration and severity of clinical signs of respiratory disease as well as rectal temperature has been observed. However, future studies of APP in equine medicine are needed before the applicability can be assessed.[85] There is an increase in APPs, particularly Hp, SAA, in chronic respiratory diseases in calves, and their evaluation could be useful in the determination of prognosis in sick calves.[86]

## Acute Phase Index

When APPs are used to assess nonhealthy animals versus healthy ones, values of single reactants are often not sensitive enough to detect a special patient or subject in a population of livestock. However, the acute phase signal obtained for an individual animal can be enhanced when the values of positive APP (rapid and slow) are combined with those of rapid and slow negative AP in an index. In starvation especially a decrease in the reactants may be expected.

$$\mathrm{Nutritional}\;\mathrm{and}\;\mathrm{acute}\;\mathrm{phase}\;\mathrm{index}\;\left(NAPI\right)\;=\;\frac{Value\;of\;a\;rapid\;positive\;APP\;\times \;Value\;of\;a\;slow\;positive\;APP}{Value\;of\;a\;rapid\;negative\;APP\;\times \;Value\;of\;a\;slow\;negative\;APP}\;$$

The index has been used as prognostic inflammatory and nutritional index (PINI) for human patients and as acute phase index (API) for cattle. NAPI enhances sensitivity and specificity of the APP to detect nonhealthy subjects in a population of normal animals. Determination of APP can help to monitor herd and individual health, especially when several acute phase variables are combined in an NAPI.[87]

### Technology to quantitatively measure proteins

Radioimmunoassay (RIA) and ELISA used for APP measurement in particular of CRP are developing methods for rapid measurements of APP values. Turbidimetry is developed for APP in the dog, horse, and for the cat. Two-dimensional electrophoresis with mass spectrometry has been shown to be applicable to animal samples with the aim to measure APRs. A protein chip has been developed for the measurement of Hp and SAA in human patients. Protein microarray methodology on slides has been proposed for APP in pigs. Preliminary experiments with a monoclonal antiporcine CRP and pig acute-phase sera using methodology as described offered the possibility to measure more than 1000 pig blood sample spots on a single slide.

Indirectly, APP formation may be measured in biopsies by methods to assess upregulation of protein synthesis [quantitative polymerase chain reaction (PCR)]. Especially the technique may be applied on samples after slaughter, or in histopathology and together with the assessment of cytokines. These technological developments may have crucial importance in the future if done rapidly, and at low costs, many samples can be handled, the APPs have a good future in diagnostics. This technique is for general assessment just as the erythrocyte sedimentation rate is used in internal medicine, but more sensitive and for special groups of patients such as hoses after castration or laprotomy.[87]

## Conclusion

Determination of animal health is important. APPs may provide an alternative means of monitoring animal health. An increased focus on the application of APP for this purpose has recently been developed. Due to a relatively short life in serum and high response in diseased animals, APP serum responses constitute a valid measure of a systemic response in diseased animals; APP serum responses constitute a valid measure of a systemic response to an initiating stimulus at the time of blood sampling. Like rectal temperature, APP levels are not suitable for establishing a specific diagnosis but can provide information about the extent of ongoing lesions in individual animals.

At the herd level, APP might be useful for determining from where the disease is spreading by providing information about the prevalence of ongoing clinical and subclinical infections indicated by the high serum concentration of selected APP and by serving as the prognostic tool, with the magnitude and duration of the APR reflecting the severity of infection. Important points to consider before using APP as markers of animal health are the possible influence of environmental factors, handling, and other types of stress in the absence of disease. APPs have their possible use as markers of domestic animal health alone or at the herd level, for the detection and as a prognostic marker of different diseases or infections. However, an international standardization of APP assays is needed before they can be applied for the systematic health monitoring in veterinary medicine.
